# Development and evaluation of a digital, community-based intervention to reduce noncommunicable disease risk in a low-resource urban setting in Malaysia: a research protocol

**DOI:** 10.1186/s43058-020-00080-y

**Published:** 2020-10-07

**Authors:** Ishu Kataria, Carrie Ngongo, Shiang Cheng Lim, Erica Kocher, Paul Kowal, Arunah Chandran, Aaron Kual, Fu-Meng Khaw, Feisul Idzwan Mustapha

**Affiliations:** 1 Center for Global Noncommunicable Diseases, RTI International, New Delhi, India; 2 Center for Global Noncommunicable Diseases, RTI International, Seattle, USA; 3 RTI International, Kuala Lumpur, Malaysia; 4 Better Health Programme Southeast Asia, Yangon, Myanmar; 5 Ministry of Health, Putrajaya, Malaysia; 6 British High Commission, Kuala Lumpur, Malaysia; 7 Public Health England, London, UK

**Keywords:** Noncommunicable diseases, Community health volunteer, Digital health, KOSPEN, Intervention, Malaysia

## Abstract

**Background:**

Noncommunicable disease burden is rising in Malaysia, accounting for 72% of all deaths. Urbanization and globalization have contributed to changing patterns of diet and physical activity, creating an obesogenic environment that increases noncommunicable disease risk, especially in low-income populations. Community-based and technological interventions can play an important role in addressing structural determinants that influence noncommunicable disease burden. The Better Health Programme Malaysia aims to co-create and develop a community-based digital intervention for low-income populations to enable community stakeholders to address obesogenic environments and improve people’s knowledge, attitudes, and practices related to noncommunicable disease risk.

**Methods:**

This quasi-experimental study will assess community member and community health volunteer knowledge, attitudes, and practices on noncommunicable disease prevention, risk factors, and health-seeking behavior in three geographical areas of Kuala Lumpur, each representing a different ethnicity (Malay, Indian, and Chinese). Assessment will take place before and after a 9-month intervention period, comparing intervention areas with matched control geographies. We plan to engage 2880 community members and 45 community health volunteers across the six geographic areas. A digital health needs assessment will inform modification of digital health tools to support project aims. Intervention co-creation will use a discrete choice experiment to identify community preferences among evidence-based intervention options, building from data collected on community knowledge, attitudes, and practices. Community health volunteers will work with local businesses and other stakeholders to effect change in obesogenic environments and NCD risk. The study has been approved by the Malaysian Ministry of Health Medical Research Ethical Committee.

**Discussion:**

The Better Health Programme Malaysia anticipates a bottom-up approach that relies on community health volunteers collaborating with local businesses to implement activities that address obesogenic environments and improve community knowledge, attitudes, and practices related to NCD risk. The planned co-creation process will determine which interventions will be most locally relevant, feasible, and needed. The effort aims to empower community members and community health volunteers to drive change that improves their own health and wellbeing. The learnings can be useful nationally and sub-nationally in Malaysia, as well as across similar settings that are working with community stakeholders to reduce noncommunicable disease risk.

**Trial registration:**

National Medical Research Register, Malaysia; NMRR-20-1004-54787 (IIR); July 7, 2020

Contributions to the literature

- The Better Health Programme in Malaysia will employ a co-creation process with communities to select and prioritizing evidence-based approaches to reduce the obesogenic environment of the urban poor.
- Research will inform the project approach, including a knowledge, attitudes, and practices (KAP) survey, a digital needs assessment, and a discrete choice experiment.
- This work will expand the evidence base around community-driven health promotion by documenting a bottom-up approach in which communities define priorities and community health volunteers work alongside local businesses to address obesogenic environments.

## Background

Noncommunicable diseases (NCDs) are the leading causes of mortality and morbidity in Malaysia, consistent with global patterns of disease burden. In Malaysia, NCDs account for an estimated 71.9% of deaths and 72.4% of lost disability-adjusted life years (DALYs) [1]. In 2019, 18.3% of Malaysian adults had raised blood glucose, 30.0% had raised blood pressure, and 50.1% were overweight or obese [2]. Global changes in patterns of diet and physical activity due to urbanization, increasingly sedentary modes of transportation and employment, and the rise of readily available, calorie-dense, and processed foods all contribute to the increasing burden of obesity and NCDs [3, 4]. Working in parallel to individual health risks, the “obesogenic environment” encompasses the physical, economic, and social aspects of an environment that influence food intake and physical activity [5]. An obesogenic environment acts as a structural determinant of health through the availability of built or natural space for physical activity and the affordability and accessibility of healthy food options. The shift toward urban population centers, where 75% of Malaysians currently live, has accelerated the burden of NCDs [6].

The rising global burden of obesity is associated with economic development and urbanization, but the impact of these socio-economic factors is not consistent across income levels [7]. Obesity and risk of NCDs are associated with low-income, low-education, and low-socioeconomic status in low- and middle-income countries [8]. As countries develop economically, the burden of obesity rises most among poorer populations [9]. People of low socioeconomic status are often disproportionately affected by negative environmental factors, contributing to the changing patterns of disparities in NCD risk factors by income [10]. Poorer populations may have limited access to affordable and healthy foods in their neighborhoods. They may work long hours or multiple jobs, leaving limited time for physical activity. Their neighborhoods may not include safe spaces for walking or other activities, a concern that can be particularly challenging in dense urban environments. In Malaysia, the poorest 40% of the population (the B40) have the highest prevalence of inadequate consumption of fruits and vegetables, and the poorest 20% of the population have the highest level of physical inactivity [11]. In a study of B40 women living in urban high-rise dwellings in Kuala Lumpur, heathy eating index scores were positively associated with income and negatively associated with an individual’s frequency of eating outside of the home, suggesting the influence of an unhealthy surrounding food environment [12]. Due in part to these increased risks, diabetes is more prevalent among the B40 than other income groups in Malaysia [11, 12].

Community-based programs have shown promise in addressing risk profiles and the obesogenic environment [13–15]. These programs have the advantage of being tailored to specific context and needs, a feature that is essential to efforts to improve the obesogenic environment and alter health behaviors [16]. Malaysia has a strong tradition of community-based health programming. Beginning in the 1980s, community health volunteers (CHVs) worked with the primary health care system to promote malaria prevention activities. The program expanded its focus to health promotion and preventive activities more broadly [17]. Other programs have successfully relied on a community-based approach to address maternal and child health and communicable diseases [18]. Since 2013 the *Komuniti Sihat Pembina Negara* (KOSPEN) program deployed a network of CHVs to conduct NCD screening and health education on a range of NCD topics, including healthy eating, active lifestyle, body weight management, smoking, and regular health screening [19, 20]. While the program is positively viewed by participating CHVs, previous evaluations have identified important limitations in its implementation, including inadequate training for volunteers and low participation and promotion among community members [19, 20]. A 2017 evaluation of KOSPEN found that CHV performance was positively associated with factors such as support and supervision by the community [21]. These results suggest that it is important to ensure that CHVs are integrated into the community to encourage their continued engagement.

Digital health technologies increasingly offer an opportunity to increase the reach and accessibility of health interventions and could complement face-to-face interactions with CHVs. In 2018, 78.0% of Malaysians reported using smartphones, including 83.8% of people in urban areas [22]. While higher-income Malaysians are more likely to use smartphones, a majority (60.9%) of Malaysians in the lowest income group are smartphone users even with monthly income of RM 1000 (USD 234) or less. Digital tools such as mobile applications, messaging services, and e-Learning are transforming how healthcare and health promotion activities are delivered. For example, the World Health Organization’s (WHO) Be He@lthy, Be Mobile Initiative is building a toolkit of population-wide digital heath prevention interventions for NCDs, including SMS messaging, mobile applications for physical activity, and digital health education [23]. In Malaysia, the KOSPEN@*Activ* program uses a mobile application that syncs to wearable fitness trackers to encourage and reward physical activity competitions between users [24]. Digital technologies are providing new tools for the health workforce in facilities and in communities. Mobile apps for CHVs include platforms for communication with community members, decision support tools, and eLearning to digitize CHV training [25–27]. eLearning for CHVs increases flexibility and reduces costs, allowing CHVs to complete training from anywhere at the time they find most convenient. A 2014 study of CHV training in South Africa estimated that a blended eLearning approach could reduce training costs by up to 42% when compared to traditional models [28]. Although digital interventions are promising, available evidence from low- and middle-income countries (LMICs) largely consists of small-scale demonstration projects [29, 30]. Digital tools must continue to be scaled and evaluated in new settings [31]. Prior qualitative research conducted in Malaysia found that individuals’ responses to web-based health promotion tools are informed by their age, gender, and socioeconomic status, underscoring the importance of designing locally specific digital tools with the target user and context in mind [32].

Given the promise of community-based interventions and value of digital health tools, the Better Health Programme (BHP) Malaysia aims to reduce risk factors for NCDs among the urban B40 through a combination of digital and in-person community-based interventions to alter the obesogenic environment. Funded by the United Kingdom’s Prosperity Fund, BHP is an initiative to reduce morbidity and premature mortality due to NCDs in order to cultivate a healthier and more productive workforce in eight countries (Brazil, Mexico, Malaysia, Myanmar, South Africa, The Philippines, Thailand, and Vietnam) [33]. In Malaysia, BHP is implemented by RTI International in partnership with PricewaterhouseCoopers. BHP Malaysia has been co-created with the Malaysian Ministry of Health and aims to address NCDs by expanding the evidence base around community-driven health promotion, improving the availability of healthier food options, and increasing B40 adoption and knowledge of healthy behaviors. During the project’s pilot phase, we will co-create packages of interventions with communities, develop appropriate digital tools, and train CHVs to partner with community organizations and businesses to implement the selected interventions. Working with CHVs, we will develop a system of supportive supervision and mechanisms that integrate and recognize CHVs within their communities. We will evaluate this pilot phase to quantify the impact of the interventions on CHV and community members’ knowledge, attitudes, and practices related to NCDs and NCD risk factors. We have built a research design into the project approach so that learnings can be consolidated, disseminated, and applied to project scale-up and sustainability.

### Objectives

We aim to evaluate a community-based intervention combining in-person and digital support to strengthen CHV capacity to interact with community stakeholders to address obesogenic environments and improve knowledge, attitudes, and practices related to NCD risk. We will compare changes in CHV and community member knowledge, attitudes, and practices in matched intervention and control communities in Kuala Lumpur (1:1). Simultaneously, we will engage with local businesses (through CHVs and digital tools) to address some of the dietary aspects of the obesogenic environment. We seek to answer several questions:

- What interventions would B40 community members prefer to enable NCD prevention?
- How can digital tools and platforms assist CHVs with their community engagement (community members and local businesses)?
- How do CHV and community member knowledge, attitudes, and health practices compare before and after the pilot implementation of selected interventions?

## Methodology

### Study design and setting

**Fig. 1:**
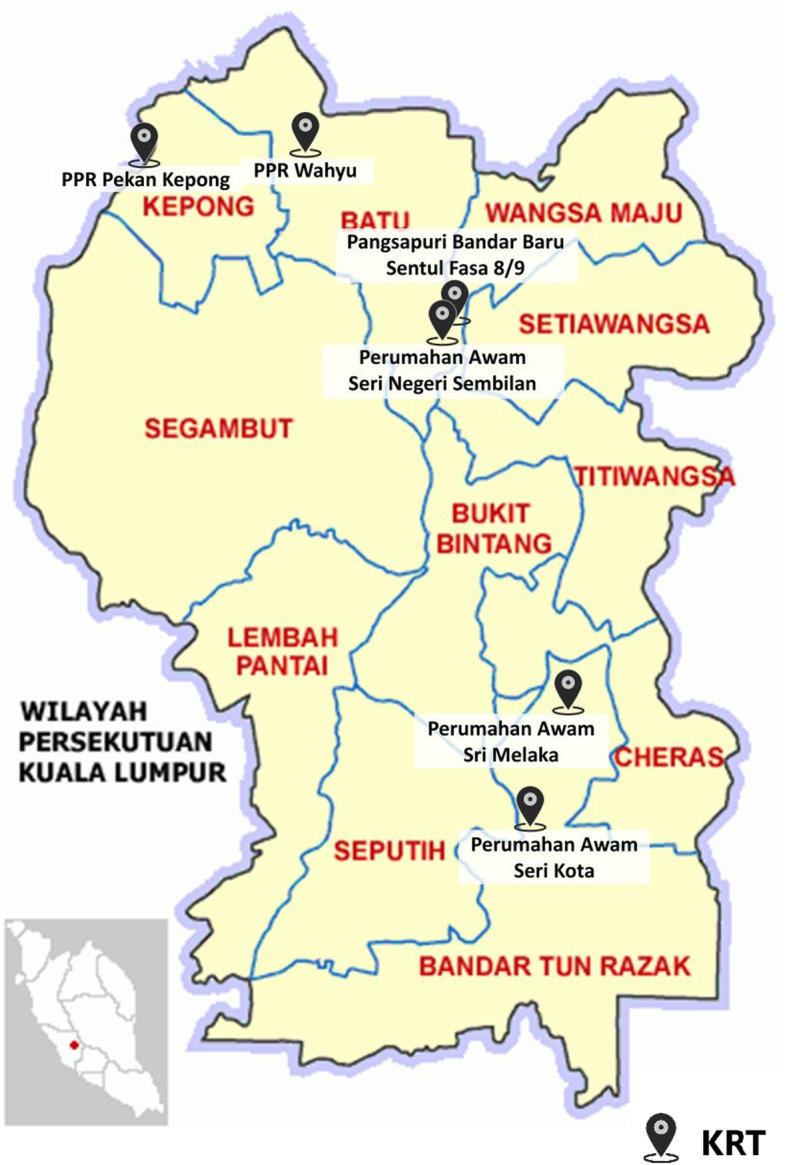
District map of Kuala Lumpur

This quasi-experimental study uses pre- and post-surveys to evaluate the effect of BHP pilot interventions, comparing intervention and control sites. The research will be conducted in the Federal Territory of Kuala Lumpur, the national capital and largest city in Malaysia. Kuala Lumpur (KL) is divided into 11 districts for administrative purposes as per the KL City Hall—city council which administers the city of Kuala Lumpur in Malaysia (Fig. 1).

Our geographical unit of focus is the *Kawasan Rukun Tetangga* (KRT), or Neighborhood Watch, which is a program under the Department of National Unity and Integration. KRTs voluntarily organize community, welfare, and education activities to strengthen community cohesion and enhance racial unity and integration. There are 307 KRTs in KL, of which 48 offer affordable housing to benefit lower income groups.

The Ministry of Health, Malaysia, has classified KL into four main districts—*Cheras, Kepong, Titiwangsa,* and *Lembah Pantai*, for ease of administration of health programs (Table 1). Four Ministry of Health District Health Offices oversee health initiatives in 11 sub-districts of KL, including the management of 13 health clinics located across the city. Each health clinic serves more than one low-income community, but most low-income KRT residents attend the same clinic.

**Table 1:** Kuala Lumpur health district classification as per the Ministry of Health, Malaysia

Main district	Cheras	Kepong	Lembah Pantai	Titiwangsa
Sub-districts	*Cheras*	*Kepong*	*Lembah Pantai*	*Titiwangsa*
	*Bandar Tun Razak*	*Segambut*	*Seputih*	*Wangsa Maju*
		*Batu*		*Bukit Bintang*
				*Setiawangsa*

*Cheras* and *Titiwangsa* are in the east while *Kepong* and *Lembah Pantai* are in the west (Fig. 1). *Cheras* and *Kepong* districts have been randomly selected for pilot implementation to ensure adequate geographic representation. Pilot KRTs will be from these two districts. Selected KRTs within the districts of *Cheras* and *Kepong* have been mapped according to a dominant ethnic group to ensure representation of Malaysia’s three main ethnic groups—Malay, Chinese, and Indian. Based on the selection criteria, a total of six KRTs (three interventions and three control groups) matched on ethnicity will be selected (Table 2). Overall, three KRTs will serve as intervention KRTs and three will serve as the control. We plan to pilot the BHP interventions for 9 months.

**Table 2:** Intervention and control KRTs

District	Dominant Ethnic Group	Intervention KRTs	Control group
Kepong	Chinese	*PPR Wahyu*	*PPR Pekan Kepong*
	Indian	*Perumahan Awam Seri Negeri Sembilan*	*Pangsapuri Bandar Baru Sentul*
Cheras	Malay	*Perumahan Awam Seri Kota*	*Perumahan Awam Seri Melaka*

### Participant recruitment and data collection

We will recruit urban B40 community members and CHVs. A total of 2880 community members across six KRTs (480 respondents per KRT) will be recruited. To be eligible to respond to the interviewer assisted survey, respondents must be 18 years or older and be able to provide informed consent. During the recruitment, we will ensure an equitable distribution of gender and all eligible age groups. We will recruit 45 CHVs (30 in intervention and 15 in control KRTs) who agree to participate for the entire duration of the pilot. Recruited CHVs must be 18 years or older, have at least completed a primary level of education, be able to read and write in Malay or English, and speak at least one of the local languages (Malay/Tamil/Mandarin/Cantonese).

### Data collection tools

#### Knowledge, attitudes, and practices (KAP) survey

The survey will assess health literacy and knowledge about NCD prevention, such as tobacco use, alcohol consumption, physical activity, diet, and health-seeking behaviors among both the community members and CHVs. It will be repeated after the 9-month pilot intervention program and will be used to evaluate the impact of the program.

#### Digital needs assessment

We will assess the digital needs of CHVs across the intervention KRTs to inform the design and adaptation of health-focused digital health tools during the development and implementation of the interventions. The survey will gauge CHV digital literacy, device access, and priorities related to digital health app format and content.

We will apply the above findings to design and conduct a discrete choice experiment (DCE). This method involves community participation in the intervention development process. Through the DCE, we will quantify the preferences of participating communities to choose between sets of potential interventions to identify their priority interventions and quantify the relative strength of their preferences for each intervention. The 1440 community members across the three intervention KRTs will be involved in conducting in-person DCE. The results will then inform the emphasis of program interventions in each KRT, facilitating a responsive program design and community ownership.

### Validation of tools

The KAP and digital needs assessment surveys are newly generated based on established evidence and tools that have been previously validated in LMIC contexts. We reviewed, for example, the Malaysia National Health and Morbidity Surveys and an NCD risk factor surveillance survey for Malaysia to aid in the development of the KAP survey. The digital health needs assessment survey was informed by best practice guidelines from the WHO and other organizations [25, 26]. Following the review of these guidelines, we adapted specific questions from existing digital literacy assessments for community health workers to create a first version of the planned survey [34].

We carried out content validation of the tools with assistance from the Ministry of Health NCD experts, who reviewed and offered recommendations to ensure the tools’ contextual relevance and acceptability. We sought support from local and international experts in NCDs and information and communication technology to help refine the tools and advise on content validity for the Malaysian context. We will pilot test the survey with a sample of community members at a different site to ensure that they are appropriately localized and clear to our B40 audience.

For identifying the interventions to be included in the DCE, we reviewed established guidelines and databases such as the WHO Best Buys, Disease Control Priorities 3rd Edition, and the World Cancer Research Fund NOURISHING Framework [35, 36]. From these sources, we assembled an initial list of evidence-based interventions that address obesogenic environments and NCD risk. We then conducted interviews with community leaders in B40 KRTs to discuss their community’s priorities for health and NCDs, their perceptions of how well existing health programming is working for their community, and their preferences and thoughts on the interventions we identified. We also consulted with local academic experts with experience in obesity prevention and nutrition policy in Malaysia. We reviewed the interventions with NCD experts at the Ministry of Health, Malaysia, to confirm their relevance, feasibility, and acceptability for B40 communities in Malaysia. Taken together, the inputs from literature, community leaders, local experts, and our team’s technical experience, we identified a number of proposed interventions to include on the DCE survey. We plan to modify these interventions, if needed, considering the results obtained from the KAP survey. We will pilot test the DCE survey with community members at a different site to ensure that the content and format are relevant, understandable, and appropriate to the B40 community.

### Engagement with local businesses

Local businesses such as food vendors, restaurants, and supermarkets are an important segment of the environment in which a community thrives. Their support and involvement in BHP are therefore critical. Our strategy of engaging local businesses will be to outline and share the BHP program benefits to the local business owners or president of local business associations by offering them opportunities for involvement, with support and introduction by KRT or KOSPEN leaders who are familiar with the local businesses landscape. Such presentations will serve as promotion efforts, set the stage for later recruitment efforts, and encourage different business groups to consider the most appropriate form of participation in the program besides building rapport with them.

Local businesses will be invited to participate in multi-stakeholder focus group discussions through assistance from CHVs. They will work with other key stakeholders from the community to help develop and co-create intervention activities that are of mutual benefit for them and the community members. Ongoing, regular communication will inform local businesses of program development and opportunities for participation in order to encourage their long-term support.

### Data analysis

We will use SPSS software to analyze the resulting data. All variables in the KAP, digital needs assessment survey, and DCE will be tested for normality by using skewness and kurtosis values. We will summarize each variable with descriptive statistics, including frequency, mean, range, percentage, and standard deviation. Since the KAP and digital needs assessment surveys will include a few open-ended questions, we will translate the responses into English, back-translate to check translation quality, and analyze them using a thematic analysis methodology. We will construct a knowledge score for each respondent which will sum up all the responses obtained through the KAP. Similarly, we will construct a summary score for participants’ digital literacy and perceptions of digital technologies.

Bivariate analyses will be performed on all outcome indicators of the KAP survey by age, gender, ethnicity, income level, and other associated individual- and community-level attributes. We will test for statistically significant differences in outcomes across groups using chi-square tests (categorical outcomes) and *t* tests (continuous outcomes) with significance thresholds of 0.05. We will measure changes in knowledge, attitudes, and practices between baseline and endline. We will then conduct a regression analysis to determine predictors of knowledge, attitudes, and practices among the study population. We do not anticipate having statistical power to conduct sub-group analyses for the digital needs assessment survey given its small sample size.

The quality of DCE responses will be assessed by evaluating the completeness and internal validity of the data. We will include several questions in the survey that will be used to confirm participant attention and comprehension, but which will be excluded from the DCE analysis [37]. These questions will include repetition of questions to confirm participants select the same option each time, or control questions in which participants are asked to choose between two sets of interventions in which one set is preferable in all of the included attributes. For example, participants may be asked to choose between one set in which all possible interventions are included and one in which no interventions are included. We will perform a conjoint analysis to model community member preferences for individual interventions, with attribute levels modeled as categorical variables. We will use a mixed-logit or random-parameter logit model. Where feasible, we will carry out sub-group analyses to identify key characteristics on which individual preferences vary, such as KRT, age, gender, ethnicity, income level, and other relevant parameters.

To report the findings from the focus group discussions with local businesses, we will use the audio-recordings to finalize the transcripts, which will be translated into English. We will conduct a thematic analysis in which we will systematically analyze the findings from each focus group discussion to identify common themes and similarities and differences amongst the target audience and will use QSR NVivo for these analyses.

### Data management

Proper data storage and security are critical to protecting data integrity, optimizing data usability, and safeguarding potentially sensitive or personally identifiable information. Data collected using electronic and data management platforms will be securely stored on a cloud-based management system with role-based access controls. If hard copy records are required, all physical copies of documents will be stored in locked file cabinets. All data will only be accessed and managed by the authorized research team. We will de-identify all the data before analysis to protect participant privacy and will only be shared with the program team and other collaborators in aggregate. We will not share any individual-level or personally identifiable data outside of the research team. We will maintain the study data for 3 years following completion.

### Quality control

BHP Malaysia partner RTI International will work closely with a fieldwork company to collect data. The fieldwork firm will complete a mandatory training of 3 days to learn the study protocol, procedures for data collection, and interactions with participants, characteristics of a good interview, scheduling interviews, handling difficult interviews, procedures for storing and sharing data, and special considerations for human subjects research and protections, including safeguards for human subjects data and how to conduct the informed consent process. Three days of in-person training will include daily practice and observation sessions. All participating field interviewers will complete the training and display competency in the study procedures, including following all safety procedures with respect to COVID-19 guidelines, prior to beginning data collection or interactions with participants.

We will work closely with the fieldwork company throughout the data collection period. As part of regular progress update meetings with the fieldwork company, we will provide notification of any changes to the protocol, consent materials, and/or data collection tools. Throughout data collection, we will monitor the quality of data collected by the fieldwork company by conducting random checks of the data being collected. A sub-sample of the data collected will be screened by the research team for its accuracy and completeness, and any necessary feedback shall be provided to the fieldwork company. A sub-sample of interviews will also be directly observed to ensure that appropriate human subjects protections and informed consent procedures are being practiced.

### Ethical considerations

We have registered the protocol for BHP Malaysia with the Malaysia Ministry of Health National Medical Research Register. We have obtained ethical approval for the BHP Malaysia from the Ministry of Health Medical Research Ethical Committee (MREC) [Approval number: NMRR-20-1004-54787 (IIR)] and plan to report any modifications to the study protocol to MREC in a timely manner. We will ensure written informed consent from all respondents before we begin data collection. Participation in the surveys is entirely voluntary. All individuals in intervention KRTs will have access to participate in the selected interventions, regardless of whether they are randomly selected to respond to the surveys. All participant responses will be confidential, securely stored, and de-identified prior to analysis.

## Discussion

The Malaysian Ministry of Health and BHP Malaysia partners propose an innovative program to improve the obesogenic environment of the urban poor. The initiative relies on volunteer CHVs to partner with business owners and other community stakeholders to lead changes that community members themselves have prioritized. This community-driven approach complements ongoing, formal primary care services, recognizing that many determinants of NCD risk occur in community environments. It builds from the recognition that change must be owned to be sustained.

While most projects determine their interventions at the outset, BHP Malaysia looks forward to community members selecting and prioritizing evidence-based approaches. This co-creation process will determine which interventions will be most locally relevant, feasible, and needed. Our project recognizes the diversity of challenges that can add up to an obesogenic environment. We hypothesize that collaboration between community stakeholders will result in increased adoption and knowledge of healthy practices that reduce NCD risk and improved the availability of healthier food options for the B40 population. Communities will define and act on their priorities.

BHP Malaysia has learned from KOSPEN and its predecessor community health efforts and builds on their model of CHVs as trusted communicators and agents of change at the KRT level. Unlike KOSPEN, BHP Malaysia does not emphasize referrals to primary health facilities for NCD screening and treatment. Its focus is squarely on community-based prevention and reduction of NCD risk. BHP Malaysia will address the obesogenic environment facing the diverse and full population of B40 residents in selected KRTs. As such, the program has the opportunity to alter diet and physical activity among community members before they might be considered in a stricter sense to be “at risk” for NCDs.

BHP Malaysia is focused on the urban poor in recognition that the poor are disproportionately affected by negative environmental factors that increase NCD risk. B40 populations have the highest rates of diabetes in Malaysia [8] and are at the greatest risk of impoverishment from the health problems caused by NCDs. They also have the lowest access to preventive and curative care. Within the B40, we will also target additional socially excluded groups, including women, diverse ethnic groups, and people with disabilities, who face further disparities and barriers to NCD prevention and health promotion activities [8, 26, 27]. We anticipate that this emphasis on the poor and socially excluded groups will address the fundamental inequities associated with NCD risk.

CHVs must be adequately supported to excel. Rising coverage of digital technology opens numerous opportunities to increase communication, education, and supportive supervision. The arrival of COVID-19 and social distancing measures has sped and highlighted the need for the flexible, remote support that digital tools can offer. We look forward to exploring how digital technology can equip CHVs in their health promotion and local advocacy efforts.

BHP Malaysia is a project with a research design. We have so much to learn from what works and what does not work in equipping communities to drive positive local change to reduce NCD risk. The KAP survey, digital needs assessment, and DCE will inform the project approach and reflect the project pilot’s effects. Regular process evaluations will reveal project challenges and strengths. BHP Malaysia anticipates adapting its work in response to lessons learnt along the journey.

Although NCDs are the leading causes of mortality and morbidity around the world, the literature includes scant evidence of effective, scaled initiatives to stem the rising tide. The innovation of BHP Malaysia is its focus on a bottom-up approach in which communities define and act on their priorities alongside engagement with local businesses which are elements of an obesogenic environment. By sharing our project approach and learnings along the way, we intend to expand the evidence base around community-driven health promotion. The relevance of this research will add value beyond national and subnational NCD policies and programs in Malaysia to similar settings that are working with CHVs to reduce NCD risk.

## Data Availability

Not applicable

## Abbreviations

NCD: Noncommunicable disease
DALY: Disability-adjusted life year
CHV: Community health volunteer
KOSPEN: Komuniti Sihat Pembina Negara
BHP: Better Health Programme
KL: Kuala Lumpur
KRT: Kawasan Rukun Tetangga
DCE: Discrete choice experiment
WHO: World Health Organization
KAP: Knowledge, attitudes, and practices
MREC: Medical Research Ethics Committee
